# 

**Published:** 1869-11

**Authors:** 


*CONTENTS:*

TRANSLATIONS:
Clinical Lectures upon Mental and Nervous Diseases, - - - 621
Amputation by the Ecraseur, - - 631
FOREIGN ITEMS, - - - - - 633
HOSPITAL REPORTS:
Cook County Hospital, - - - 636
SOCIETY REPORTS:
Intemperance as a Disease, - - 638
ORIGINAL COMMUNICATIONS:
Remarkable Case of Somnambulism, - 650
Biliary Calculi, - - - - - 655
EDITOR'S BOOK TABLE, - - - - 657
Hammond on Sleep.
Laurence's Hand-Book Ophthalmic Surgery.
Thompson on Stricture of Urethra, &c.
Pamphlets.
EDITORIAL:
Improvement in THE JOURNAL, - 669
On Specialties, - - - - - 670
Ozone, - - - - - - - 670
Arrearages, - - - - - - 671
LOOT:
On Certain Influences of Schools Injurious to Health. By Prof. Virchow, 671
Ozæna Cured, - - - - - - 678
Diphtheria, - - - - - - - 678
Common Catarrh, - - - - - 678
Neuralgia, - - - - - - - 679
Pertussis, - - - - - - 679
Sore Nipples, - - - - - 679
Trismus Nascentium, - - - 679
Impetigo — Scabies — Diarrhœa of Children, - - - - - - - 680
Primitive Chancres, - - - 680
Hæmoptysis—Phthisis, - - - 681
Varicose Ulcers, - - - - 681
Drowning, Restoration from - - 681
Cutaneous Affection Caused by the Use of Bromide of Potassium, - - 683
Leucorrhœa in a Young Girl, - - 683
Digitalis as a Diuretic, - - - 684
Management of After Pains, - - 684
MEDICAL JOURNAL ADVERTISER, - - 685

